# Extensible lateral approach versus sinus tarsi approach for sanders type II and III calcaneal fractures osteosynthesis: a randomized controlled trial of 186 fractures

**DOI:** 10.1186/s13018-024-05345-z

**Published:** 2025-01-03

**Authors:** Amr A. Fadle, Ahmed A. Khalifa, Peter Mamdouh Shehata, Wael EL-Adly, Ahmed Ekram Osman

**Affiliations:** 1 Orthopaedic Department, Assiut Faculty of Medicine, Assiut University Hospital, Assiut University, Kasr Elini Street, Number 7, P.O. Box 110, Assuit, 71515 Egypt; 2 Orthopaedic Department, Qena Faculty of Medicine and University Hospital, South Valley University, Qena, Egypt

**Keywords:** Calcaneal fractures, Extensible lateral approach, Sinus tarsi approach, complications

## Abstract

**Aims:**

Which is the best extensile lateral (ELA) or sinus tarsi (STA) approach for osteosynthesis displaced intraarticular calcaneal fracture (DIACF) is still debatable. The current RCT’s primary objective was to compare the complications incidence after open reduction and internal fixation of DIACFs through STA vs. ELA. The secondary objectives were the differences in intraoperative radiation exposure, time to fracture union, functional and radiological outcomes.

**Methods:**

Between August 2020 and February 2023, 157 patients with Sanders type II and III fractures were randomly assigned to either ELA (81 patients with 95 fractures) or STA (76 patients with 91 fractures). The primary outcome was the incidence of complications. The secondary outcomes were Böhler’s and Gissane angles angle, fracture union, and American Orthopaedic Foot and Ankle Society (AOFAS) score.

**Results:**

No statistical differences between both groups regarding basic demographic data, injury characteristics, and fracture classification; however, patients in the STA group were operated upon significantly earlier (4.43 ± 7.37 vs. 7 ± 6.42 days, *p* = 0.001). STA’s operative time was significantly shorter (55.83 ± 7.35 vs. 89.66 ± 7.12 min, *p* < 0.05), and no statistical difference regarding intraoperative radiation exposure. The time to fracture union was significantly shorter in STA (6.33 ± 0.8 vs. 7.13 ± 0.7 weeks, *p* = 0.000). Skin complications (superficial or deep infection) and Subtalar osteoarthritis were significantly higher in ELA (18.9% vs. 3.3%, *p* = 0.001) and (32.6% vs. 9.9%, *p* = 0.001), respectively. The radiological parameters were significantly better in STA postoperatively and at the last follow up. The AOFAS scores were significantly better in STA (83.49 ± 7.71 vs. 68.62 ± 7.05, respectively, *p* = 0.000).

**Conclusion:**

During osteosynthesis of Sanders type II and III DIACFs, STA is superior to ELA in terms of operating earlier, shorter operative time, fewer complications, and better radiological and functional outcomes.

## Introduction

Tarsal bone fractures represent about 1 to 2% of all skeletal injuries, while calcaneus fractures are the most commonly occurring, constituting about 60% of foot fractures [1, 2]. Although nonoperative management options were suggested to result in outcomes comparable to operative intervention for managing displaced intraarticular calcaneal fractures (DIACF) [3, 4], open reduction and internal fixation (ORIF), aiming at anatomical fracture reduction and joint congruency restoration, is considered by some surgeons as the management of choice as these fractures involve the articular surface and occur mostly in younger populations [4–6].

The extensile lateral approach (ELA) has been the workhorse with satisfactory exposure and outcomes for ORIF of DIACF [7, 8]. However, owing to the extensive soft tissue dissection, it entails various complications, mainly soft tissue related, such as skin necrosis, wound dehiscence infection, and sural nerve injury, leading to increased hospital admissions and the need for secondary surgical intervention [7, 9, 10].

To avoid such complications, various strategies were introduced, including percutaneous reduction and fixation [1, 11], and utilizing minimally invasive (MI) approaches such as the limited posterior [12], and sinus tarsi (STA) approach [10, 13, 14].

The STA is appealing due to its less traumatic and soft tissue-preserving nature. It allows early surgical intervention; furthermore, surgeons can obtain proper fracture reduction and fixation, leading to better outcomes and fewer complications than other approaches [14–17].

Studies comparing both approaches showed controversial results regarding which is better and safer [18–20]; moreover, well-designed studies are relatively scarce, as mentioned in a recent systematic review and meta-analysis by Attenasio et al. evaluating studies comparing ELA and STA included 21 studies, and only four were randomized controlled trials (RCTs) [15]. This emphasizes the need for more and larger RCTs to reach conclusive evidence regarding the superiority of one approach over the other; previous authors raised this concern as well [14, 21, 22].

The current RCT’s primary objective was to evaluate the incidence of complications after calcaneal fractures osteosynthesis (ORIF) through STA compared to ELA. The secondary objectives were reporting the differences in intraoperative radiation exposure, time to fracture union, functional and radiological outcomes. We hypothesized that STA would result in a lower incidence of complications compared to ELA and involve more intraoperative radiation exposure but would not demonstrate superior functional or radiological outcomes.

## Patients and methods

### Trial design

This prospective randomized controlled trial (RCT) was performed at the trauma unit at Assuit University Hospital (a level-one North African trauma center) between August 2020 and February 2023. Our institution’s Ethical Committee approved the study (IRB no: 17101516), and it was registered at ClinicalTrials.gov (NCT04509895). The study was carried out according to the Helsinki Declarations, and written informed consent was obtained from all study participants. The CONSORT recommendations for reporting RCTs were followed (supplementary file1) [23].

### Participants (inclusion and exclusion criteria)

We included adult patients (≥ 18 years old) who presented to our trauma unit with recent closed DIACF, classified as either type II or III per Sanders’ classification [24], completed at least one year of follow up, and approved to participate in the study. In contrast, patients < 18 years old, those who presented late after the initial trauma, fractures classified as type I or IV, extra-articular fractures (tongue type fractures), presented with open fractures, did not complete the follow up period, patients with associated other skeletal injuries preventing or interfering with the postoperative rehabilitation protocol and those who refused to participate in the study were excluded.

### Randomization and blinding

Over the study period, 172 patients were eligible for inclusion; they were randomly allocated (allocation ratio 1:1) to either of the treatment groups (Group A: ELA and Group B: STA) using a computer-generated system. The random distribution details were concealed in a sealed envelope. Patients were sequentially numbered and given to the head nurse, who informed the surgeon regarding the allocation before the operation. All surgeries were performed by fellowship-trained surgeons familiar with both approaches. By default, surgeons, assistants, and assessors could not be blinded regarding the type of surgical intervention. However, the assessor was blinded to functional outcomes assessment by asking the patient to wear socks so that the assessor would not notice the surgical scar.

**Fig. 1:**
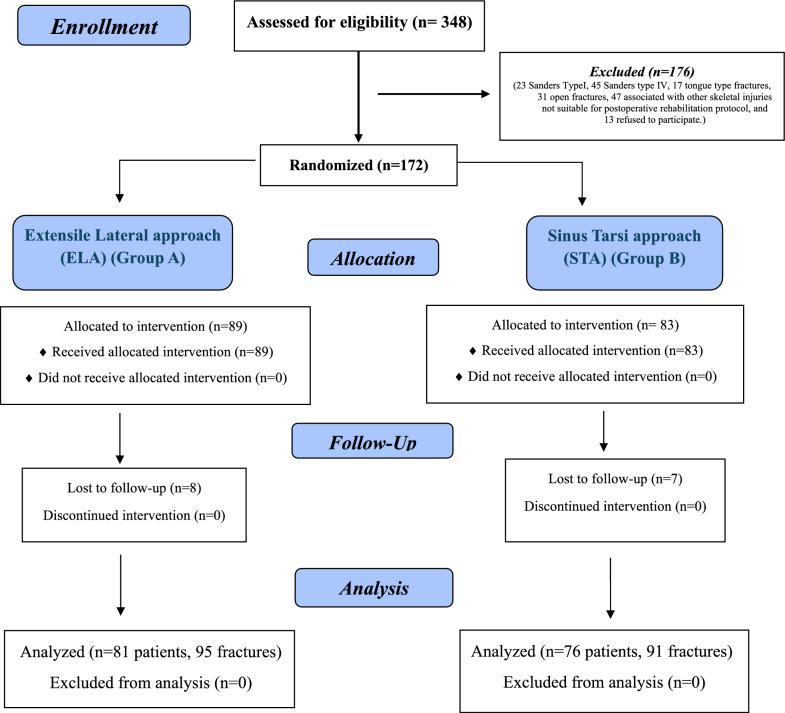
Consolidated standards of reporting trials (CONSORT) flow diagram of the patient’s enrollment

During the study period, 89 patients were treated through the ELA and 83 through STA; however, 15 patients were lost during the follow up, leaving 157 patients for final inclusion [81 in Group A (95 fractures, 14 bilateral, and 67 unilateral) and 76 in Group B (91 fractures, 15 bilateral and 61 unilateral)], details of allocation are shown in Fig. 1.

### Intervention and surgical technique

#### Preoperative assessment

On admission, all patients were managed according to the ATLS protocol. After patients’ clearance, calcaneus fractures were assessed by obtaining axial and lateral ankles plain radiographic views of the and computed tomography (CT) scans to determine Sanders fracture classification. Bulky Jones dressing was applied with limb elevation, and the skin condition was followed up until edema resolved. Skin wrinkles on the lateral aspect of the calcaneus were seen, denoting good skin condition and suitability of surgical intervention.

#### Operative details

For either approach, we followed the surgical steps described previously in the literature [13, 19]. All patients were operated upon under spinal anesthesia except for 24 patients who had concomitant spinal fractures and had general anesthesia. A thigh tourniquet was applied in all patients, and the patient position was either prone or lateral decubitus according to surgeon preference. Under fluoroscopic control, a Steinmann pin was initially inserted in the calcaneal tuberosity to apply traction and correct varus deformity in all patients.

#### Sinus tarsi approach

**Fig. 2:**
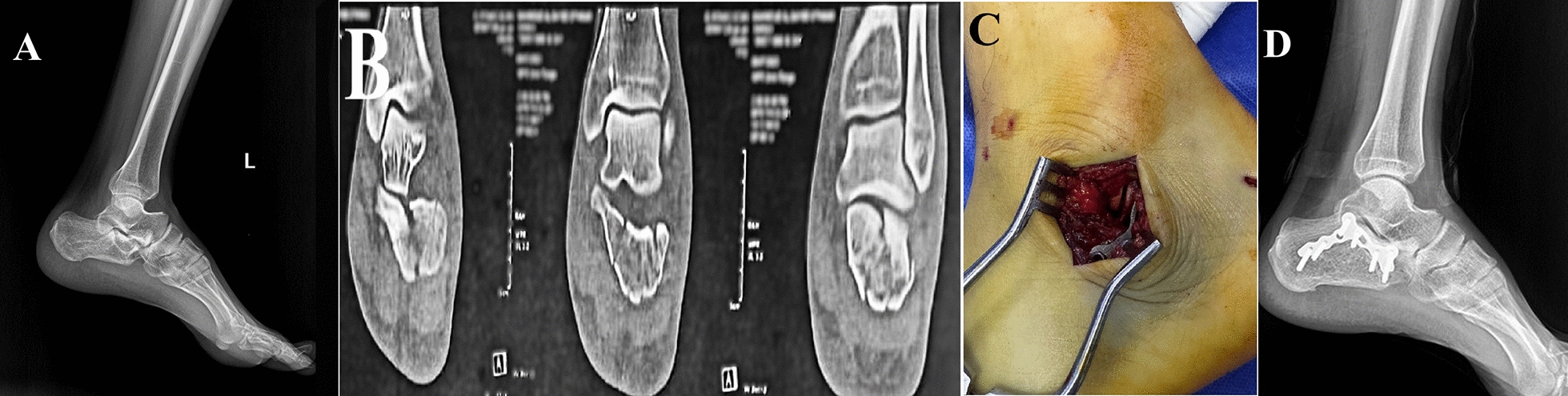
Male patient 35 years old having Sanders type III fracture treated by open reduction and internal fixation through sinus tarsi approach (STA). **A** Preoperative radiograph showing intraarticular calcaneal fracture with apparent joint depression. **B** Preoperative computed tomography showing Sander’s type III fracture. **C** Intraoperative demonstration of the STA. **D** Plain radiographs at one-year follow-up showed fracture union and maintained articular surface

A 4–5 cm incision was made over the sinus tarsi, carefully dissecting to avoid injury to nearby tendons and nerves. The fascia was incised, and the posterior facet was exposed, elevated, and temporarily fixed with K-wires. Two fixation tools were used, at the surgeon’s discretion, either 6.5 mm cannulated screws or sinus tarsi locked plate, in the screws group of the sinus tarsi approach. When screws were used, 6.5 mm screws were applied to secure reduction, maintain calcaneus length and height, while a 4.5 mm partially threaded cannulated screw was used as a sustentacular screw. Screws length ranged from 35 to 50 mm. If a plate was chosen, it was used mainly to secure fracture reduction and buttress the lateral wall blowout (Fig. 2).

#### Extensile lateral approach

**Fig. 3:**
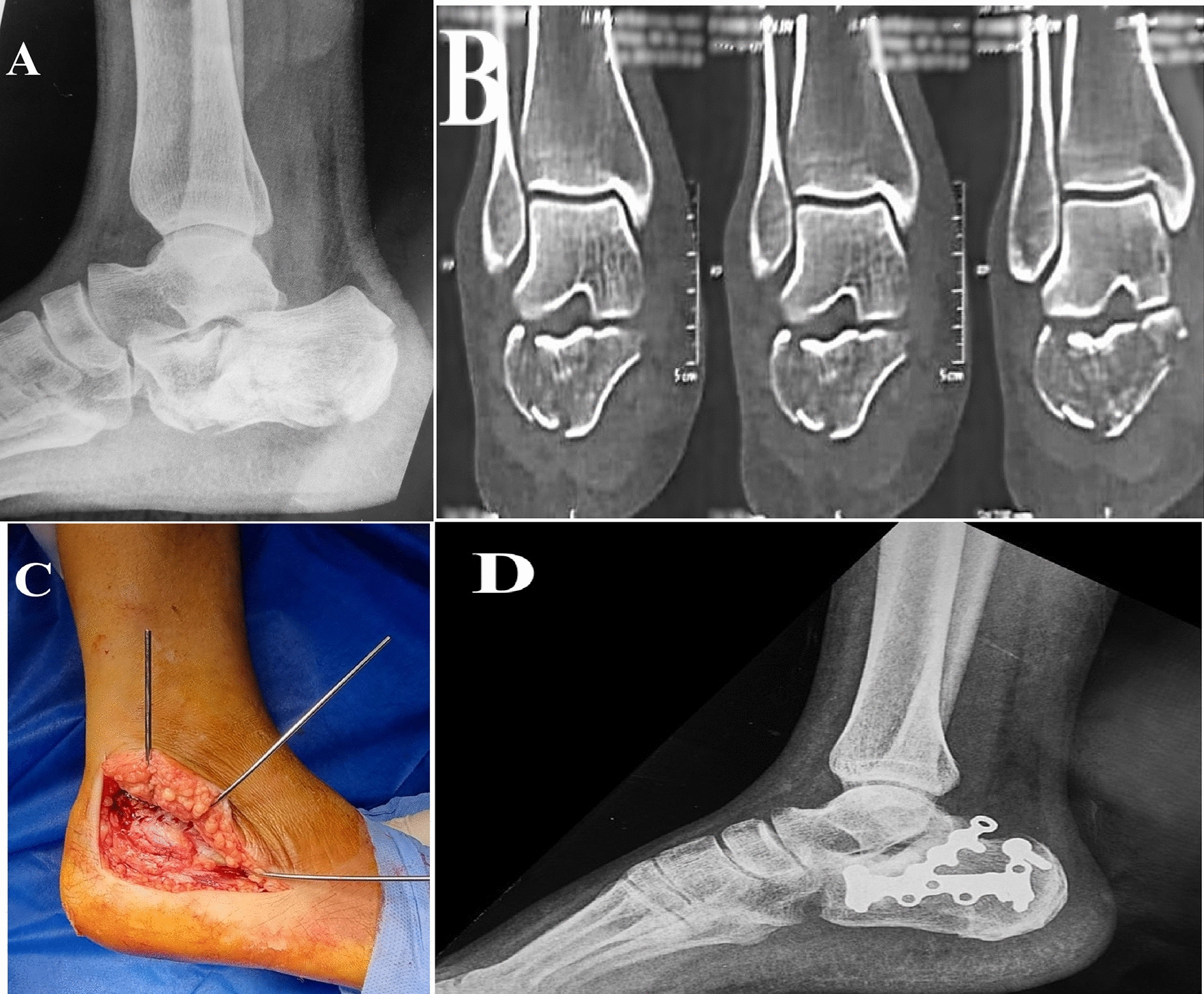
Female patient 41 years old having Sanders type III fracture treated by open reduction and internal fixation through extensible lateral approach (ELA). **A** Preoperative radiograph showing intraarticular calcaneal fracture with apparent joint depression. **B** Preoperative computed tomography showing Sanders type III fracture. **C** Intraoperative demonstration of the ELA. **D** Plain radiographs at one-year follow-up showed fracture union and maintained articular surface

Surgery was carried out through a lateral L-shaped incision. A full-thickness flap was created, including the peroneal tendons and sural nerve. The subtalar joint was exposed, and retraction K wires were placed into the talus and fibula lateral process. The lateral wall fragment was either reflected or temporarily removed as needed. The depressed posterior facet is elevated and fixed to the constant fragment by a sustentacular screw. A low-profile lateral calcaneal plate was applied (in some cases, a small DCP was used). Soft tissue closure involves a two-layer closure over a drain, using absorbable stitches for subcutaneous tissue and interrupted Allgöwer-Donati stitches for skin, ensuring no excessive tension on the incision (Fig. 3).

### Postoperative follow up protocol

Immediately postoperatively, patients were placed in a well-padded splint, intravenous antibiotics were given during the hospital stay only, and patients were monitored for compartment syndrome development.

On the second operative day, the splint was changed to check the wound and skin condition, and the patient was discharged. Patients were asked to follow up in the clinic after one week for initial wound evaluation and two weeks for suture removal. After that, patients were placed in a short-leg, well-padded splint for six weeks and encouraged to do ankle and subtalar motion as tolerated.

Partial weight bearing was allowed starting six weeks postoperatively, and full weight bearing was allowed after radiological evidence of union.

Patients were asked to present to the hospital at any time if they felt symptoms suggesting wound infection, such as local itching sensation or fever, or symptoms suggesting compartment syndrome development, such as severe pain, tingling, or paresthesia.

### Outcomes assessment

A. Surgical wound complications were reported as either minor (superficial infection or marginal necrosis not requiring surgical intervention) or major (wound dehiscence, deep wound infection requiring surgical intervention or hardware removal).
B. Other complications occurring during the follow-up period, including non-union, reduction loss, and development of subtalar arthritis (classified according to Kellgren and Lawrence Grading System (KLGS)) [25, 26], were reported.
C. Immediate postoperative radiographic images (lateral and axial views) were obtained to judge the fracture reduction and implant positioning; these were repeated at six weeks postoperatively, three months, six months, and then at 12 months. Böhler and Gissane angles were measured serially to detect any failure of reduction where poor or loss of fracture reduction was defined as displacement of 2 mm and loss of ≥ 5° of the Böhler angle, respectively. The preoperative, immediate postoperative, and at least after one year follow up measurements were reported.
D. Fracture union was evaluated clinically (no swelling, painless weight bearing, and no tenderness over the calcaneus) and radiologically (if there were bony bridging in radiographic images, no loss of reduction, and maintained implant position); non-union was defined if the above criteria were not achieved after six months postoperatively [27, 28].
E. Functional outcomes were assessed using the American orthopaedic foot and ankle society ankle/hindfoot score (AOFAS) and were reported at least after one year of follow up [29, 30].
F. The need for revision surgery, hardware removal, and any secondary procedures during the follow up were reported.

### Statistical analysis

Statistical analysis was performed using SPSS v26 (IBM Inc., Armonk, NY, USA). Quantitative variables were presented as mean and standard deviation (SD), and a comparison between both groups was made utilizing an unpaired Student’s t-test. Qualitative variables were presented as frequency and percentage (%), and both groups were compared using the Chi-square or Fisher’s exact test when appropriate. *P* value < 0.05 was considered statistically significant.

## Results

*Patients’ Demographics and fracture-related characteristics* There were no differences between both groups regarding basic demographic data, injury side, fracture classification, mode of trauma, and associated injuries; however, patients in ELA presented significantly later compared to patients in STA, 7 ± 6.42 versus 4.43 ± 7.37 days, respectively, *p* = 0.001, details are reported in (Table 1).

**Table 1:** Baseline data, injury characters, and operative details

	Group A, ELA (n = 95 fractures in 81 patients, 14 bilateral and 67 unilateral)	Group B, STA (n = 91 fractures in 76 patients, 15 bilateral and 61 unilateral)	*P* value
*Baseline data*			
Age (years)*	34.11 ± 12.3	33.3 ± 12.84	0.571
Gender^†^			
Male	65 (80.2%)	63 (82.9%)	0.839
Female	16 (19.8%)	13 (17.1%)	
Smoking^†^	31 (38.3%)	27 (35.5%)	0.421
DM^†^	4 (4.9%)	2 (2.6%)	0.473
Occupation^†^			
Unemployed	25 (30.9%)	21 (27.6%)	0.573
Student	10 (12.3%)	8 (10.5%)	
Manual worker	41 (50.6%)	43 (56.6%)	
Others	5 (6.2%)	4 (5.3%)	
*Injury characteristic*			
Mode of trauma^†^			
Fall from height	61 (75.3%)	52 (68.4%)	0.291
Fall on ground	6 (7.4%)	9 (11.8%)	
Fall downstairs	11 (13.6%)	10 (13.2%)	
Road traffic accident	3 (3.7%)	5 (6.6%)	
Affected side^†^			
Right	53 (55.8%)	43 (47.3%)	0.078
Left	42 (44.2%)	48 (52.7%)	
Fracture classification^†^			
Sander II	40 (42.1%)	45 (49.5%)	0.31
Sander III	55 (57.9%)	46 (50.5%)	
Associated fractures^†^	(Fracture spine: 17, Fracture pelvis: 5, Degloved skin over elbow joint: 1)^‡^	20 (26.3%) (Fracture spine: 15, Fracture pelvis: 4, fracture humerus greater tuberosity: 1)^‡^	0.718
Mean time from trauma to surgery*	7 ± 6.42	4.43 ± 7.37	**0.001**
*Operative time and radiation exposure*			
Operative time (minute)*	89.66 ± 7.12	55.83 ± 7.35	**0.023**
Radiation shots (number) (equivalent radiation exposure in mSv)*	12.66 ± 4.1 (2.532 ± 0.82 mSv)	21.33 ± 9.11 (4.27 ± 1.82 mSv)	0.485

Bold values indicating statistical significance (P value < 0.05)

DM, diabetes mellites

^*^Data are presented as mean ± SD

^†^Data are presented as frequency (percentage)

^‡^ Associated fracture required conservative management

*Operative details* Operative time was significantly shorter in STA compared to ELA, 55.83 ± 7.35 versus 89.66 ± 7.12 min, (*p* = 0.023). Although the radiation exposure was higher in STA, the difference was insignificant, 4.27 ± 1.82 versus 2.532 ± 0.82 mSv (*p* = 0.485). No cases in STA required intraoperative conversion to an ELA.

**Fig. 4:**
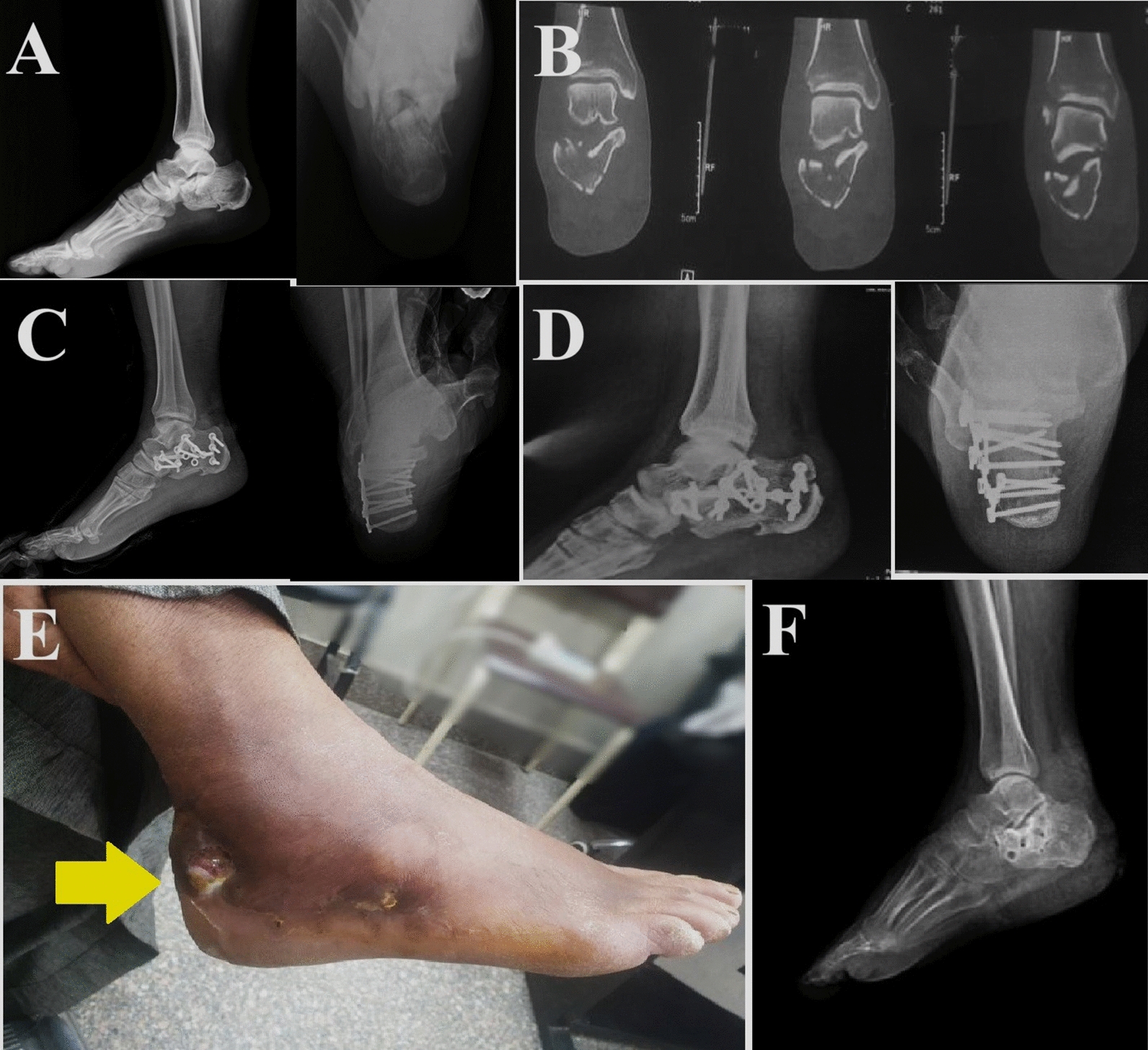
Male patient, 63 years old, presented with fractured left calcaneus, Sanders III, osteosynthesis by open reduction, and internal fixation through extensible lateral approach (ELA). **A** and **B** Preoperative images. **C** Immediate postoperative, and **D** Last follow up radiographs. **E** After 7 months of follow up, he presented with a sinus discharging pus (yellow arrowhead). **F** Debridement and metal removal were performed

*Complications* Skin complications (superficial or deep infection) were significantly higher in ELA, 18 (18.9%) versus 3 (3.3%), respectively, *p* = 0.001. Two (2.2%) in STA and 11 (11.6%) from ELA were superficial infections treated with daily dressings and antibiotics. In contrast, seven (7.4%) in ELA and one (1.1%) in STA had deep infections that required surgical debridement and metal removal (Fig. 4).

**Fig. 5:**
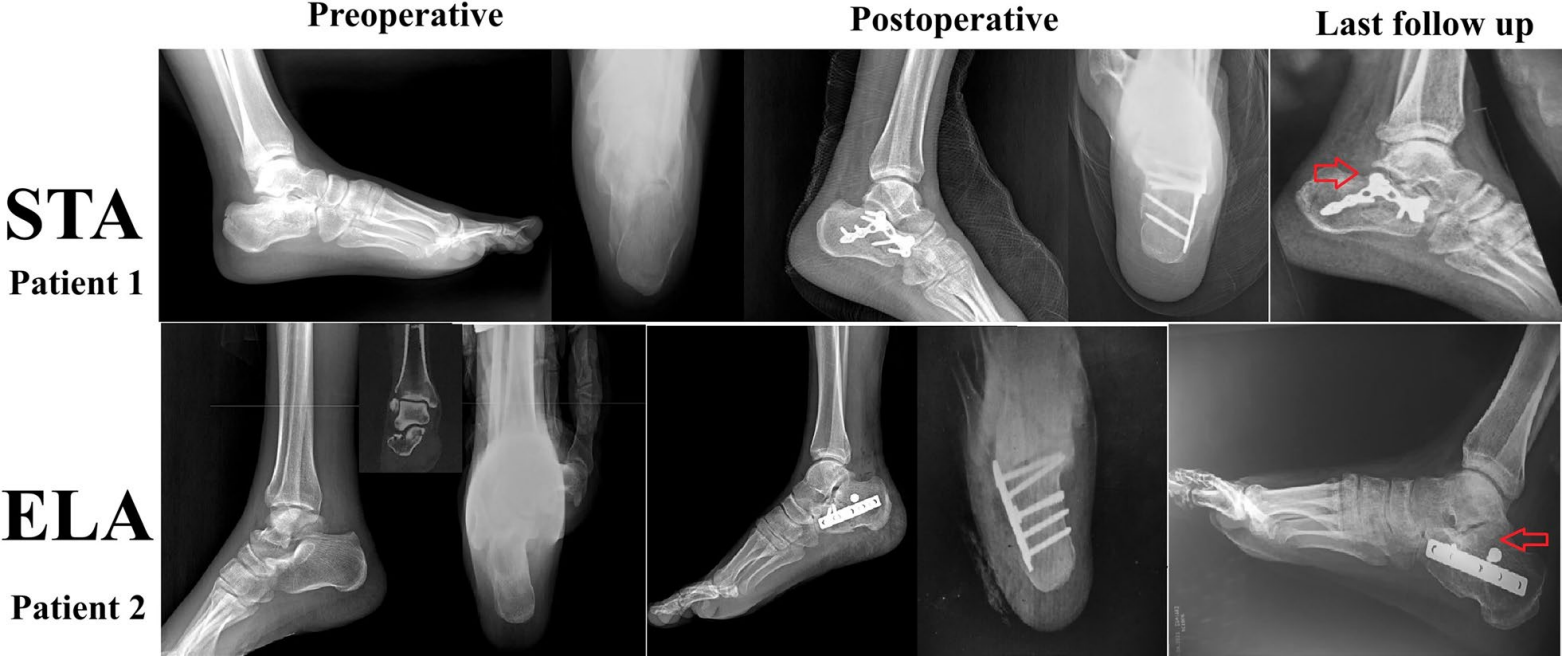
Examples of subtalar arthritis (indicated by red arrowheads) occurring with both approaches. Patient 1, male 38 years old, presented with Sanders II left calcaneus fracture, osteosynthesis through sinus tarsi approach (STA); at the last follow up (10 months), he developed subtalar arthritis. Patient 2, Male 22 years, presented with fracture right calcaneus, Sanders II, osteosynthesis through extensible lateral approach (ELA); at the last follow up (8 months), he developed subtalar arthritis

Regarding other reported complications, the most commonly occurring was subtalar osteoarthritis, which was significantly higher in ELA, 31 (32.6%) versus 9 (9.9%), respectively, *p* = 0.001; the details of complications and the secondary surgical interventions were reported in Table 2 (Fig. 5).

**Table 2:** Complications, radiological and functional outcomes

	Group A, ELA (n = 95 fractures)	Group B, STA (n = 91 fractures)	*P* value
*complications*			
Skin infection^†^	18 (18.9%) (11 superficial, 7 deep)	3 (3.3%) (2 superficial and 1 deep)	**0.001**
Sub-talar arthritis^†^	31 (32.6%) (12 KL1, 8 KL2, 4 KL3, 7 KL4)	9 (9.9%) (3 KL1, 2 KL2, 3 KL3, 1 KL4)	**0.001**
Sural nerve palsy^†^	2 (2.1%)	1 (1.1%)	0.976
Metal irritation^†^	7 (7.4%)	2 (2.2%)	0.397
Peroneal tendinitis^†^	3 (3.2%)	0 (0%)	0.431
*Secondary procedures*			
Revision^†^	1 (1.1%)	1 (1.1%)	0.976
Metal removal^†^	9 (9.5%)	1 (1.1%)	**0.001**
Sub-talar arthrodesis†	11 (11.6%)	3 (3.3%)	**0.001**
*Clinical outcomes*			
AOFAS at one year*	68.62 ± 7.05	83.49 ± 7.71	**0.000**
Time to union (weeks)*	9.13 ± 0.7	8.33 ± 0.8	**0.000**
*Radiological outcome*			
Bohler angle*			
Preoperative	9.14 ± 5.5	11.45 ± 6.83	0.16
Postoperative	21.42 ± 4.11	28.45 ± 4.77	**0.001**
Last follow-up	17.78 ± 3.35	24.29 ± 5.64	**0.001**
P-value (between preoperative and last follow up)	**0.001**	**0.001**	
Gissane angle*			
Preoperative	156.12 ± 6.73	150.92 ± 7.098	**0.001**
Postoperative	138.32 ± 13.98	132.53 ± 5.24	**0.001**
Last follow-up	143.66 ± 6.329	137.8 ± 6.07	**0.001**
*P*-value (between preoperative and last follow up)	**0.001**	**0.001**	

Bold values indicating statistical significance (P value < 0.05)

AOFAS, American Orthopaedic Foot and Ankle Society; KL, Kellgren and Lawrence grading system

^*^Data are presented as mean ± SD

^†^Data are presented as frequency (percentage)

*Radiological outcomes* The time to achieve fracture union was significantly shorter in STA, 8.33 ± 0.8 versus 9.13 ± 0.7 weeks, respectively, *p* = 0.000. The radiological parameters were not different preoperatively between both groups; however, all the parameters were significantly better in STA than in ELA postoperatively and at the last follow up. For both groups, radiological parameters at the last follow up were significantly better than preoperative values (Table 2).

*Functional outcomes* At one year follow up, the AOFAS scores were significantly better in STA, 83.49 ± 7.71 versus 68.62 ± 7.05, respectively, *p* = 0.000.

## Discussion

Comparing STA to ELA while osteosynthesis DIACF was evaluated in various studies, systematic reviews, and metanalysis; although multiple reports suggested the superiority of STA, it seems that the results are controversial, and the dominance of one approach over the other is still inconclusive [9, 17, 22, 28, 31, 32].

The current RCT offered a larger number of patients (186 Sanders type II and III fractures) compared to previously reported studies (where patient numbers ranged from 21 to 120) [10, 15]. We found significantly better functional and radiological outcomes with the STA. The incidence of complications, mainly infection and subtalar arthritis, was substantially lower while utilizing the STA. Furthermore, we were able to operate on patients with the STA earlier than ELA. So, our hypothesis was partially accepted.

The overall complications in the current study were higher with the ELA compared to STA, specifically skin infection and subtalar arthritis. The skin might be at greater risk for complications during ELA as the lateral calcaneal artery (LCA), which is the main blood supply to the lateral aspect of the calcaneus, could be injured while performing the vertical incision of the ELA as it passes midway between the fibula and tendoachilles [33, 34]. Such possible injury is avoided while operating through the STA.

In a systematic review by Attenasio et al., including 2086 patients (1129 ELA vs. 957 STA), the studies included all four Sanders fracture types (88.8% of which were type II and III). They reported significantly higher (2.82 times) wound complications (necrosis, infection, and dehiscence) in the ELA compared to STA [15]. Zeng et al. carried out a systematic review and meta-analysis by including eight RCTs (495 patients presented with Sanders II and III fractures) comparing ELA to MI approaches (only four RCTs including 251 patients reported direct comparison between ELA and STA); the authors reported significantly lower complication incidence with MI approaches compared to ELA (RR = 5.10, 95% CI [2.37, 10.95], *P* < 0.0001) [10]. On the contrary, in an RCT by Park et al. comparing the ELA vs. STA while treating 64 patients presented with Sanders type 2 fractures (32 patients in each group). The incidence of complications was higher in the ELA group (12.5% vs. 0%); however, this difference was insignificant (*p* = 0.113) [21].

We reported a relatively high incidence of subtalar arthritis, which was significantly higher in ELA than STA (32.6% versus 9.9%, *p* = 0.000); it is worth noting that 20 (64.5%) out of the 31 subtalar arthritis in ELA patients were either KL grade 1 or 2. In a retrospective cohort study by Yeo et al. comparing ELA (60 patients) versus STA (40 patients) for managing Sanders II and III calcaneal fractures, they reported insignificant difference in subtalar stiffness incidence between both approaches, 7.5% vs. 8.3% for STA and ELA groups respectively, *p* = 0.458 [35]. However, a higher rate of subtalar arthritis, up to 77%, was reported in a study by Schindler et al., where most patients were treated through an ELA [36]. Angthong et al. could not define factors leading to early subtalar arthritis (incidence of 21.1% at a mean follow up of 14.7 months) after surgical management of calcaneal fractures [37]. However, Rao et al. defined Sanders fracture type (90% of fractures included in their study were types II and III) as a predictor for subtalar arthritis development (OR = 4.04, 95%CI = 1.43–11.39, *p* = 0.0084), with a greater percentage occurring with types III and IV [38].

We believe that the higher incidence of subtalar osteoarthritis in the ELA group is not merely a matter of surgical approach; however, several factors could contribute. During ELA, the surgeon could mobilize the fracture fragments more vigorously than when operating through a limited approach such as STA, which could jeopardize the blood supply of these fragments. Second, the longer time passed between injury and surgery in the ELA group could have affected the articular cartilage blood supply and nutrition by hematoma compression [39–41]. Third, possible chondral surface injury during the initial trauma, which could not be evaluated in plain radiographs or CT scans [38, 42]. Last, it could be surgeon-induced due to poor fracture reduction as indicated by worse immediate postoperative radiological outcomes in the ELA group compared to STA [40].

For the sural nerve injury-related symptoms, in the current study, no difference was found between both groups (*p* = 0.976); the same results were reported by Park et al. [21]. From an anatomical standpoint, the sural nerve and its branches are at risk in both approaches [21, 43]. During the ELA, the main sural nerve trunk crosses the incision vertical limb; even if it was clearly identified, its lateral calcaneal branch is at risk while elevating the lateral full-thickness flap [43]. While during SAT, the sural nerve’s main trunk or its anastomotic branches could be injured at the distal incision level, and some surgeons advised performing a horizontal incision parallel to the sole to prevent such injury [44, 45].

Supporters of the ELA suggest that it offers enough visualization for better fracture reduction compared to the limited exposure provided by MI approaches, including the STA [46, 47]; however, in the systematic reviews by Zeng et al. [10], and Attenasio et al. [15], the authors showed no significant difference regarding the radiological outcomes indicating the ability of STA to offer visualization with subsequent optimum fracture reduction comparable to the ELA. In the current study, we reported significantly better radiological outcomes in the STA compared to the ELA group immediately postoperatively and at last follow up, as indicated in Table 2. Although it was expected that owing to better exposure with the ELA, the radiological outcomes immediately postoperatively should be better or even similar to those obtained with a limited exposure approach (STA), the contradiction found in the current study could be attributed to the longer time passed between injury and surgery in the ELA group, which could have led to difficulty in fracture fragment mobilization to obtain anatomical reduction. Furthermore, it was noticed that the Bohler angle values were reduced, and the Gissane angle values were increased in both groups in the last follow-up, which could be attributed to the influence of subtalar joint arthritis development.

Contrary to the previously mentioned results, Busel et al. [47] evaluated the quality of fracture reduction in 83 Sanders II and III fractures, where 36 were treated through STA vs. 47 through ELA; the authors reported better overall fracture reduction in the ELA group (*P* = 0.02), normal Bohler angle was achieved more often in ELA group, 91.5% vs. 77.8% (*P* < 0.001). At the same time, neither approach had any difference regarding the Gissane angle (*P* = 0.5).

The literature (individual studies and systematic reviews) provided controversial results regarding the differences in the functional outcomes between both approaches. Some reported better outcomes with the STA [10, 15, 16]. In contrast, others suggested no differences between both approaches [31, 32, 46]. In the current study, at a minimum of 12 months follow up, functional scores per AOFAS were significantly better with STA than ELA (68.62 ± 7.05 vs. 83.49 ± 7.71, respectively, *p* = 0.000), which could be explained by the fact that higher incidence of wound complications and subtalar arthritis in the ELA group could have compromised the functional outcomes. Park et al. [21] reported that AOFAS scores were significantly better in the STA group only at six months follow up (*p* = 0.021) but not at one year (*p* = 0.200). Attenasio et al. reported significantly better AOFAS scores in STA than ELA by a mean of 2.31 points [15]. Zeng et al. reported significantly better AOFAS scores with MI approaches compared to ELA (MD =  − 1.78 95% CI [-3.22, 0.34], *P* = 0.02) [10].

To be noted, STA offered further advantages: first, the shorter time from injury to surgery; as with such an MI approach, surgeons are not waiting for complete clearance of the edema and the appearance of wrinkle signs [15]. The time from injury to surgery was 5.24 ± 4.2 versus 3.35 ± 3 days for ELA and STA, respectively (*p* = 0.044), according to Park et al. [21]. In a series of 121 patients (55 ELA vs. 74 STA), Lim et al. reported a longer time to definitive fixation in the ELA compared to STA, 23 days versus 14 days, respectively (*P* = 0.0001) [14]. Furthermore, Attenasio et al. reported a significantly shorter time to surgery for STA than ELA, with a mean difference of 2.37 days [15]. The same notice was reported in the current study where 7 ± 6.42 days vs. 4.43 ± 7.37 days passed from injury to surgery in the ELA and STA groups, respectively (*p* = 0.001).

Second is the shorter operative time, which could contribute to lower complication rates (as it entails less tissue trauma, less tourniquet time, and less tissue retraction time). The shorter operative time for the STA was evident in the current study (55.83 ± 7.35 min vs. 89.66 ± 7.12 min (*p* = 0.02). This was reported by many authors as well; Zeng et al. reported that the operative time was significantly shorter with MI approaches compared to ELA ((MD = 24.96, 95% CI [22.41, 27.50], *P* < 0.00001) [10]. According to Attenasio et al., the operative time was significantly longer with the ELA than STA by a mean difference of 17.58 min [15].

Third, STA had fewer secondary re-operations incidents. Attenasio et al. reported a re-operation incidence of 1.85 times higher in ELA than STA [15]. In the current study, we had a higher re-operative incidence in ELA patients, mainly for removing irritating hardware and performing subtalar arthrodesis.

One significant limitation of the current study is the lack of detailed cost analysis differences between both approaches, considering the complications, the need for secondary surgeries with subsequent hospital admissions, longer hospital stays, and the added burden of patient work absence. This point was evaluated by Li et al. [48], who found slightly higher costs with ELA. Second, we did not compare the radiological parameters between the injured and the non-injured side, which would have added to the quality of radiological outcomes. Third, no CT scan was obtained during follow-up visits to evaluate the reduction quality better. Fourth, the radiographic assessment of subtalar osteoarthritis was performed using non-weight-bearing radiographs, which could have underestimated the amount of joint collapse. Fifth, we should have investigated the factors affecting the incidence of complications rather than the surgical approach itself. Sixth, the unblinded assessment of radiological and functional outcomes could have led to some bias in reporting the results. Last, we reported outcomes at a maximum of 12 months follow up, which could be considered a relatively deficient follow up; however, according to Eckstein et al., 82% of patients with surgically managed calcaneal fractures reached a steady state from a clinical standpoint after a mean of 14 months postoperatively [49], so, reporting results at 12 months could reflect on those of longer follow-ups.

In conclusion, when osteosynthesis (ORIF) Sanders type II and III calcaneal fractures, STA is superior to ELA in terms of less complication incidence and better radiological and functional outcomes. Furthermore, it provided the benefits of shorter time from injury to surgery and lower operative time. However, further cost-effectiveness analysis to support the superiority of STA over ELA is warranted.

## Data Availability

No datasets were generated or analysed during the current study.

## Declarations

**Ethical approval.**

This article does not contain any experimental studies with human participants or animals performed by any of the authors, and the ethical committee of our institution approved it: Faculty of Medicine, Assiut University, Egypt ((IRB No.:17101516) (Telephone, Fax: + 20882332278, ethics-committee12@yahoo.com, http://afm.edu.eg). Registration of clinicaltrials.gov (ID: NCT04509895).

## Study setting

Trauma unit, Orthopaedic Department, Assiut University Hospital, Assiut, Egypt.
